# Diagnostic value of the 2011 International Federation for Cervical Pathology and Colposcopy Terminology in predicting cervical lesions

**DOI:** 10.18632/oncotarget.24074

**Published:** 2018-01-08

**Authors:** Aiping Fan, Chen Wang, Liqin Zhang, Ye Yan, Cha Han, Fengxia Xue

**Affiliations:** ^1^ Department of Gynecology and Obstetrics, Tianjin Medical University General Hospital, Tianjin 300052, China

**Keywords:** cervical intraepithelial neoplasia, colposcopy, International Federation for Cervical Pathology and Colposcopy Terminology, squamous intraepithelial lesion, terminology

## Abstract

**Objective:**

To evaluate the diagnostic accuracy of the 2011 International Federation for Cervical Pathology and Colposcopy (IFCPC) colposcopic terminology.

**Methods:**

The clinicopathological data of 2262 patients who underwent colposcopy from September 2012 to September 2016 were reviewed. The colposcopic findings, colposcopic impression, and cervical histopathology of the patients were analyzed. Correlations between variables were evaluated using cervical histopathology as the gold standard.

**Results:**

Colposcopic diagnosis matched biopsy histopathology in 1482 patients (65.5%), and the weighted kappa strength of agreement was 0.480 (P<0.01). Colposcopic diagnoses more often underestimated (22.1%) than overestimated (12.3%) cervical pathology. There was no significant difference between the colposcopic diagnosis and cervical pathology agreement among the various grades of lesions (P=0.282). The sensitivity, specificity for detecting high-grade lesions/carcinoma was 71.6% and 98.0%, respectively. Multivariate analysis showed that major changes were independent factors in predicting high-grade lesion/carcinoma, whereas transformation zone, lesion size, and non-stained were not statistically related to high-grade lesion/carcinoma.

**Conclusions:**

The 2011 IFCPC terminology can improve the diagnostic accuracy for all lesion severities. The categorization of major changes and minor changes is appropriate. However, colposcopic diagnosis remains unsatisfactory. Poor reproducibility of type 2 transformation zone and the significance of leukoplakia require further study.

## INTRODUCTION

Cervical cancer is the most common malignancy among women in China. Because of the widespread implementation of cervical cancer screening with cervical cytology and/or human papilloma virus (HPV) testing, colposcopy has become more frequently used globally. The use of colposcopy has led to a beneficial reduction in the number of unnecessary blinded biopsies and conization procedures as well as in the frequency of cauterization therapy for cervical erosions. Although colposcopic assessment continues to advance and evolve, universally accepted colposcopic diagnostic criteria and terminology are lacking. Most investigators believe that the traditional Reid colposcopic index (RCI) [1] is simple and easy to use, and it is widely applied. However, some scholars believe that the standard method of evaluating color, vessels, and margins of a colposcopic lesion (i.e., grading) to determine the most serious lesion for biopsy has been questioned and traditional grading systems do not accurately assess lesion severity because colposcopic impression alone is unreliable for diagnosis [2]. Most reports have shown a perfect agreement between the colposcopic impression and histology of only 32% −37%, with the weighted kappa strength of agreement of only 0.20-0.26. The agreement of the colposcopic diagnosis and cervical pathology within one grade has been reported to be only 75% −77%, and nearly one - third of high-grade squamous intraepithelial lesions (HSIL) were under graded [3–5]. The goal of clinical management is to identify and treat high-grade disease to decrease the risk of developing invasive cancer. Therefore, the role of colposcopy and biopsy is to identify cervical intraepithelial neoplasia (CIN), especially HSIL. In 2011, the International Federation for Cervical Pathology and Colposcopy (IFCPC) released a new terminology system (the 2011 IFCPC version of the colposcopic terminology, following the 1975, 1990, and 2002 versions of the colposcopic terminology system) and proposed replacing all previous versions of the colposcopic terminology [6]. Although similar to the 2002 version, the new IFCPC terminology is more comprehensive and reclassified some abnormal colposcopic findings on the basis of recent research. In addition, new colposcopic criteria and descriptions of vaginal lesions were added. The aims of the present study were to 1) determine the accuracy of colposcopic diagnosis using the new IFCPC colposcopic terminology; 2) assess the significance of individual colposcopic findings in the new IFCPC colposcopic terminology system; and 3) analyze each specific colposcopic characteristic included in the new IFCPC colposcopic terminology system.

## RESULTS

The data of 2262 patients were included in the analysis. The mean patient age was 41.3 years (41.3 ± 11.6 years). The agreement of the colposcopic diagnosis and cervical biopsy pathology was perfectly matched in 1482 patients (65.5%), and the weighted kappa strength of agreement was 0.480 (p < 0.01). Moreover, agreement within one grade (referring to the same grade or to a difference of only one grade) was observed in 2232 patients (98.7%), and the weighted kappa strength of agreement was 0.980 (p < 0.01). In those patients who were histologically diagnosed as normal or benign, low grade squamous intraepithelial lesions (LSIL), HSIL, or cancer, the agreement between the colposcopy diagnosis and pathological diagnosis was 63.7% (440/691), 66.4% (624/940), 67.4% (366/543), and 59.1% (52/88), respectively. There was no significant difference among the four groups (χ^2^=3.813, p = 0.282). The colposcopic diagnosis was overestimated in 279 patients (12.3%, 279/2262) and underestimated in 501 patients (22.1%, 501/2262). Within two grades (referring to the same grade or to a difference of two grades) or more, 7 patients (0.3%, 7/2262) were overestimated and 23 patients (1.0%, 23/2262) were underestimated (Table 1). The accuracy of colposcopy for the detection of LSIL or more (LSIL+) and HSIL or more (HSIL+) is shown in Table 2.

**Table 1:** Agreement between colposcopic diagnoses and cervical histological diagnoses (%)

Colposcopic diagnosis	Cervical biopsy pathology				Total
	Normal/benign	LSIL	HSIL	MIC/invasive cancer	
Normal/benign	440(63.7)	290(30.8)	19(3.5)	0(0)	749
LSIL	244(35.3)	624(66.4)	156(28.7)	4(4.5)	1028
HSIL	6(0.9)	26(2.8)	366(67.4)	32(36.4)	430
MIC/invasive cancer	1(0.1)	0(0)	2(0.4)	52(59.1)	55
Total	691(100)	940(100)	543(100)	88(100)	2262

LSIL, low-grade squamous intraepithelial lesion; HSIL, high-grade squamous intraepithelial lesion; MIC, micro invasive cervical cancer.

**Table 2:** Accuracy of colposcopic diagnosis in distinguishing cervical pathology at different cutoffs

	Cutoff for colposcopic diagnosis	
	Any lesion (low-grade/high-grade/carcinoma) vs. healthy cervix	High-grade lesion/carcinoma vs. healthy cervix/low-grade lesion
Sensitivity (95% CI)	80.3% (78.4-82.3%)	71.6% (68.1%-75.1%)
Specificity (95% CI)	63.7% (60.1%-67.3%)	98.0% (97.3%-98.7%)
Positive predictive value (95% CI)	83.4% (81.5%-85.3%)	93.2% (91.0%-95.4%)
Negative predictive value (95% CI)	58.7% (55.2%-62.3%)	89.9% (88.5%-91.3%)
Youden index (95%CI)	44.0% (39.9%-48.1%)	69.6% (66.0%-73.2%)

Of the 2262 patients, 340 patients underwent conization or hysterectomy. The agreement of the colposcopic diagnosis and final histopathologic diagnosis was perfectly matched in 225 patients (66.2%) (Table 3). In those patients who were histologically diagnosed as normal or benign, LSIL, HSIL, or cancer, the agreement between the colposcopy diagnosis and final pathological diagnosis was 100.0% (0/0), 72.7% (8/11), 68.9% (199/289), and 45.0% (18/40), respectively. There was a significant difference among the four groups (χ^2^=9.153, p = 0.010). Although there is no colposcopic overestimate, 115 patients (33.8%, 115/340) were underestimated.

**Table 3:** Agreement between colposcopic diagnoses and final histological diagnoses (%)

Colposcopic diagnosis	Final histological diagnosis				Total
	Normal/benign	LSIL	HSIL	MIC/invasive cancer	
Normal/benign	0(0)	3(27.3)	4(1.4)	0(0)	7
LSIL	0(0)	8(72.7)	86(29.7)	2(5.0)	96
HSIL	0(0)	0(0)	199(68.9)	20(50.0)	219
MIC/invasive cancer	0(0)	0(0)	0(0)	18(45.0)	18
Total	0(0)	11(100)	289(100)	40(100)	340

LSIL, low-grade squamous intraepithelial lesion; HSIL, high-grade squamous intraepithelial lesion; MIC, micro invasive cervical cancer.

Overall, 1005 cases (44.4%, 1005/2262) were classified as type 2 transformation zone (TZ), 887 (39.2%, 887/2262) as type 1 TZ, and 370 (16.4%, 370/2262) as type 3 TZ. Among 370 cases classified as type 3 TZ, the age of 243 patients (65.7%, 243/370) was > 50 years, whereas the frequency of this TZ type was low in women aged 30-50 years (31.9%, 118/370) and rare in those aged < 30 years (2.4%, 9/370). The most frequently reported TZ type in women aged < 30 years was type 1 (70.6%, 283/401). Among 1005 cases classified as type 2 TZ, 665 patients (66.2%, 665/1005) were aged 30-50 years. The linear trend test showed a correlation between TZ types and the three age groups (χ^2^ = 534.324, p< 0.01).

Of 2262 patients, 1955 patients demonstrated abnormal colposcopic findings on < 30% of the visible cervix (86.4%, 1955/2262), 148 patients had colposcopic abnormalities on 30%-67% of the visible cervix (6.5%, 148/2262), and 159 patients had abnormalities on > 67% of the visible cervix (7.0%, 159/2262). The linear trend test showed a correlation between the size of the lesion and pathological diagnosis: the larger the size of the lesion, the more serious the disease (χ^2^ = 466.107, p< 0.01).

Correlation between abnormal colposcopic findings and cervical histological diagnoses is shown in Table 4. With regard to *minor changes*, the incidence of a thin acetowhite epithelium was the highest (65.4%, 1479/2262), followed by fine punctation (48.5%, 1096/2262) and fine mosaic (37.2%, 842/2262). The sensitivity, specificity, positive predictive value (PPV), negative predictive value (NPV), and Youden index for the detection of LSIL are shown in Table 5.

**Table 4:** Correlation between colposcopic findings and cervical histological diagnoses (%)

Colposcopic finding	Cervical biopsy pathology				Total
	Normal/benign	LSIL	HSIL	MIC/invasive cancer	
**Minor changes**					
Thin acetowhite epithelium	518(35.0))	790(53.4)	168(11.4)	3(0.2)	1479(100)
Fine mosaic	210(24.9)	406(48.3)	220(26.1)	6(0.7)	842(100)
Fine punctation	337(30.8)	559(51.0)	193(17.6)	7(0.6)	1096(100)
**Major changes**					
Dense acetowhite epithelium	18(3.3)	68(12.6)	374(69.0)	82(15.1)	542(100)
Coarse mosaic	0(0)	4(2.4)	131(79.9)	29(17.7)	164(100)
Coarse punctation	1(0.4)	18(7.5)	186(77.2)	36(14.9)	241(100)
Sharp border	3(6.2)	2(4.2)	31(64.6)	12(25.0)	48(100)
Inner border sign	0(0)	7(25.0)	19(67.9)	2(7.1)	28(100)
Ridge sign	2(1.9)	7(6.7)	77(73.3)	19(18.1)	105(100)
**Non specific**					
Non-stained	523(27.0)	808(41.6)	522(26.9)	87(4.5)	1940(100)
Leukoplakia	3(20.0)	4(26.7)	8(53.3)	0(0)	15(100)
**Suspicious for invasion**					
Atypical vessels	1(1.3)	1(1.3)	21(26.5)	56(70.9)	79(100)
**Miscellaneous finding**					
condyloma	7(18.9)	26(70.3)	4(10.8)	0(0)	37(100)
polyp	93(95.8)	2(2.1)	2(2.1)	0(0)	97(100)

LSIL, low-grade squamous intraepithelial lesion; HSIL, high-grade squamous intraepithelial lesion; MIC, micro invasive cervical cancer.

**Table 5:** Accuracy of colposcopic minor changes in predicting low-grade lesion

Minor changes	Sensitivity (95% CI)	Specificity (95% CI)	Positive predictive value (95% CI)	Negative predictive value (95% CI)	Youden index (95%% CI)
Thin aceto-white epithelium	84.0% (81.7%-86.4%)	47.9% (45.2%-50.6%)	53.4% (50.9%-56.0%)	80.8% (78.1%-83.6%)	31.9% (28.4%-35.5%)
Fine mosaic	43.2% (40.0%-46.4%)	67.0% (64.5%-69.5%)	48.2% (44.8%-51.6%)	62.4% (59.9%-64.9%)	10.2% (6.2%-14.3%)
Fine punctation	59.5% (56.3%-62.6%)	59.4% (56.7%-62.0%)	51.0% (48.0%-54.0%)	67.3% (64.6%-70.0%)	18.8% (14.7%-23.0%)

Colposcopic findings graded as major changes had a high predictive value for HSIL/carcinoma. The sensitivity, specificity, PPV, NPV, and Youden index for the detection of HSIL+ for *major changes* are shown in Table 6. The two new colposcopic criteria (inner border sign and ridge sign) also had a high predictive value for HSIL+. The specificity and PPV of the inner border sign for HSIL+ was 99.6% (1624/1631) and 75.0% (21/28), respectively. The specificity and PPV of the ridge sign for HSIL+ was 99.4% (1622/1631) and 91.4% (96/105), respectively.

**Table 6:** Accuracy of colposcopic major changes in predicting high-grade lesion/carcinoma

Major changes	Sensitivity (95% CI)	Specificity (95% CI)	Positive predictive value (95% CI)	Negative predictive value (95% CI)	Youden index (95% CI)
Dense aceto-white epithelium	72.3% (68.6%-75.7%)	94.7% (93.5%-95.8)	84.1% (80.8%-87.1%)	89.8% (88.3%-91.2%)	67.0% (63.3%-70.7%)
Coarse punctation	35.2% (31.5%-39.1%)	98.8% (98.2%-99.3%)	92.1% (88.0%-95.2%)	79.8% (77.9%-81.5%)	34.0% (30.3%-37.8%)
Coarse mosaic	25.4% (22.0%-28.9%)	99.8% (99.4%-99.9%)	97.6% (93.9%-99.3%)	77.6% (75.7%-79.3%)	25.1% (21.7%-28.5%)
Sharp border	6.8% (4.8%-8.8%)	99.7% (99.4%-100%)	89.6% (80.9%-98.2%)	73.4% (71.6%-75.3%)	6.5% (4.5%-8.5%)
Ridge sign	15.2% (12.5%-18.3%)	99.4% (99.0%-99.7%)	91.4% (84.4%-96.0%)	75.2% (73.3%-77.0%)	14.7% (11.7%-17.5%)
Inner border sign	3.3% (2.1%-5.0%)	99.6% (99.1%-99.8%)	75.0% (55.1%-89.3%)	72.7% (70.8%-74.5%)	2.9% (1.5%-4.3%)

Of the non-specific signs, non-stained lesions were very common (85.8%, 1940/2262) and had a high sensitivity and NPV, but it was unsatisfactory for the specificity, PPV and Youden index. Although iodine-stained lesions had a high specificity, PPV and NPV, the sensitivity was very low (27.3%). Among 15 patients (0.7%, 15/2262) with leukoplakia, LSIL was found in 4 patients, and HSIL in 8 patients. Leukoplakia had a considerable PPV for HSIL (53.3%, 8/15).

Invasive cancers were rare and found in only 88 patients (3.9%, 88/2262). However, some colposcopic findings classified as *suspicious for invasion*, such as atypical vessels had a considerable PPV for HSIL+ (97.5%, 77/79).

Several colposcopic findings classified as *miscellaneous findings* had a low PPV (condyloma: 10.8%, 4/37; polyp: 2.1%, 2/97) for HSIL+. Among 37 patients with condyloma, LSIL was found in 26 patients, and HSIL in 4 patients. Among 97 patients with a polyp, LSIL was found in 2 patients, and HSIL in only 2 patients.

Some colposcopic findings in predicting LSIL/HSIL+ are summarized in Tables 7 and 8. On the basis of univariate analysis, factors such as TZ type (p=0.019 and p=0.001 for LSIL and HSIL+, respectively), lesion size (p=0.002 and p=0.000 for LSIL and HSIL+, respectively), colposcopic minor/major changes (both p=0.000), and no-stained lesions (both p=0.000) were all linked with the diagnostic accuracy of colposcopy for predicting LSIL and HSIL+. However, leukoplakia (p=0.999 and p=0.822 for LSIL and HSIL+, respectively) did not correlate with the diagnostic accuracy of colposcopy for predicting LSIL and HSIL+. On the basis of multivariate analysis with binary logistic regression, lesion size (p=0.036) and colposcopic minor changes (p=0.000) were significant independent factors for predicting LSIL, and only colposcopic major changes (p=0.000) were a significant independent factor in predicting HSIL/carcinoma.

**Table 7:** Colposcopic findings in predicting low-grade lesion

Colposcopic findings	n	Discordant (n=317)	Concordant (n=623)	Univariate analysis OR (95% CI)/p	Binary logistic regression OR (95% CI)/p
Transformation zone type				0.782(0.637-0.960)/0.019	1.161(0.920-1.465)/0.209
1	424	134	290		
2	422	138	284		
3	94	45	49		
Percentage of cervix encompassed by the lesion				2.775(1.446-5.329)/0.002	2.047(1.049-3.991)/0.036
< 30%	887	312	575		
30%~67%	34	2	32		
> 67%	19	3	16		
Minor changes				3.083(2.506-3.793)/0.000	3.145(2.505-3.949)/0.000
≤1	233	144	89		
≥2	707	173	534		
Non-stained lesions				0.390(0.269-0.566)/0.000	0.658(0.432-1.002)/0.051
Yes	808	247	561		
No	132	70	62		
Leukoplakia				827311037.174(0.000-∞)/0.999	2379464167.734(0.000-∞)/0.999
Yes	4	0	4		
No	936	317	619		

**Table 8:** Colposcopic findings in predicting high-grade lesion/carcinoma

Colposcopic findings	n	Discordant (n=213)	Concordant (n=418)	Univariate analysis OR (95% CI)/p	Binary logistic regression OR (95% CI)/p
Transformation zone type				1.478(1.166-1.873)/0.001	1.347 (0.992-1.828)/0.056
1	178	75	103		
2	309	102	207		
3	144	36	108		
Percentage of cervix encompassed by the lesion				2.571(1.976-3.345)/0.000	1.079 (0.781-1.489)/0.645
< 30%	391	176	215		
30%~67%	104	20	84		
> 67%	136	17	119		
Major changes				5.373(4.144-6.965)/0.000	5.070 (3.853-6.673)/0.000
≤1	319	182	137		
≥2	312	31	281		
Non-stained lesions				0.179(0.069-0.465)/0.000	0.383(0.103-1.428)/0.153
Yes	609	197	412		
No	22	16	6		
Leukoplakia				0.847(0.201-3.580)/0.822	1.012(0.164-6.251)/0.990
Yes	8	3	5		
No	623	210	413		

We also analyzed HPV genotyping data. Of the 2262 patients, 62 were not tested for HPV, 284 were negative for HPV, 23 were positive for Hybrid Capture II test, and 1893 were positive for HPV genotyping test. Single high-risk HPV (HR-HPV) infections were found in 993 of 1893 patients (52.4%), single low-risk HPV (LR-HPV) infections were found in 72 of 1893 patients (3.8%), and multiple infections ( multiple HR-HPV infections, 440 cases; HR-HPV+ LR-HPV multiple infections, 375 cases; LR-HPV multiple infections, 13 cases) were present in 828 of 1893 positive samples (43.7%). Most of the multiple infections consisted of two HPV types (58.9%, 488/828), whereas the rest had three to eight HPV types. A high prevalence of single HR-HPV infection was found among HSIL (56.1%, 291/519) and micro invasive cervical cancer (MIC) / invasive cancer (66.7%, 54/81). Significant differences (χ^2^=19.935, p=0.000) between single HR-HPV infections and multiple HR-HPV infections were observed regarding the histology findings on colposcopically directed biopsy. HPV Type 16 was identified most frequently (33.9%, 642/1893), followed by types 52(17.0%, 321/1893), 58 (16.1%, 304/1893), 56(9.2%, 174/1893), 33(8.7%, 164/1893), and 18 (8.6%, 162/1893). The prevalence of HPV type 16 increased with the grade of cervical disease, as defined by biopsy-histological diagnosis: 18.2% (167/910) in LSIL, 51.6% (268/519) in HSIL, and 81.5% (66/81) in cervical carcinoma. On the basis of univariate analysis (HR, 0.567; 95%CI, 0.397-0.808; p=0.002) and multivariate analysis (HR, 0.667; 95%CI, 0.454-0.979; p=0.039), the presence of HPV type 16 was correlated with cervical HSIL+, and it was an independent predictor factor for cervical HSIL+ development. Although HPV types 18, 58, 52, and 33 were also identified frequently in HSIL+, presence of HPV types 18, 58, 52, and 33 was not associated with this risk.

## DISCUSSION

Colposcopic examination involves the systematic evaluation of the lower genital tract. The diagnostic criteria for colposcopy continue to improve and evolve [1, 6, 9, 10]. However, the literature regarding the accuracy of colposcopy is contradictory, and no uniform standard has been accepted. The correlation between the colposcopic impression and histology remains poor [11–14].

We analyzed the applicability of the new IFCPC colposcopic terminology for predicting cervical lesions. The study results showed that the agreement of the colposcopic diagnosis and cervical biopsy pathology was perfectly matched in 65.5% of lesions, further, the agreement of the colposcopic diagnosis and final pathology was perfectly matched in 66.2% of lesions, with findings significantly better than those of Baum et al. and Massad et al (perfect agreement, 32-37%) [3, 4]. Tatiyachonwiphut et al. [13] reported an exact agreement of 57.9%, one grade agreement of 94.1%, and a weighted kappa strength of agreement of 0.494, which were comparable to the results of our study. However, the study lacked a standard criterion or scoring system for colposcopic examination. Li et al. [14], using the 2011 IFCPC colposcopic terminology, reported findings comparable to those of our study; however, their sample size was small. Mousavi et al. and Durdi et al. [11, 15] reported a higher rate of perfect agreement (77.3% and 85.8%, respectively) and a substantial level of correlation (κ = 0.74 and κ = 0.73, respectively) using the RCI scoring system, but the sample sizes were small and HR-HPV testing was not done. In addition, the RCI method depends on the severity of disease, and the predictive accuracy increases with the advancing severity of the lesion. Our study showed that there were no significant differences between the colposcopic diagnosis and cervical pathology agreement among the various grades of lesions (P = 0.282), further suggesting that the accuracy of colposcopic diagnosis using the IFCPC terminology was stable and did not vary with lesion severity.

In the present study, the colposcopic diagnosis more frequently underestimated (22.1%) than overestimated (12.3%) the cervical biopsy pathology. For those patients who underwent conization or hysterectomy, although there is no colposcopic overestimate, 115 patients (33.8%, 115/340) were underestimated. An overestimated colposcopic diagnosis leads to an unnecessary cervical biopsy, whereas an underestimated diagnosis leads to missed diagnoses, possibly high-grade lesions/carcinomas. The primary task for colposcopists is to improve the accuracy of colposcopic diagnosing HSIL+ and prevent the growth of undiagnosed lesions.

In the present study, we found that the colposcopic examination has a high sensitivity(80.3%) and is capable of differentiating a healthy cervix from any cervical lesion in 80.3% of the cases; however, the specificity was low (63.7%), probably leading to over-examination and thereby increased patients cost. Some studies have reported that such over-examination causes the patient to undergo an unnecessary cervical biopsy. We also found that colposcopy had a 71.6% sensitivity for the detection of HSIL+, similar to that reported in previous studies (30%-91.3%) [9, 13–17]. Moreover, the specificity for detecting HSIL was high (98.0%), also comparable to that previously reported (79% - 96.5%) [13–15]. A high specificity lowers the misdiagnosis rate, which is important with regard to clinical management because of the significant differences in the clinical management of LSIL and HSIL. The PPV of colposcopy to diagnose HSIL+ was 93.2%, which is also comparable to the findings of previous reports [9, 13–15]. The NPV of HSIL was 89.9%, again comparable with previous studies (83.8%-93.5%) [13–15]. On the basis of these findings, if a cervical biopsy specimen was obtained from every abnormal area detected during colposcopy, 28.4% of patients would benefit from early detection and treatment of HSIL+, and only 2.0% of patients would have undergone an unnecessary cervical biopsy.

Precise identification of the squamocolumnar junction and evaluation of the TZ are crucial steps during colposcopy. The 2011 IFCPC colposcopic terminology confirmed the classification of the TZ as an obligatory terminology. Our study showed a linear trend between the TZ type and three age groups, with type 3 TZ found more commonly in women > 50 years old and type 1 TZ found more frequently in women < 30 years old. Further, in the present study, univariate analysis revealed that TZ type was linked to the diagnostic accuracy of colposcopy. However, binary logistic regression analysis revealed that TZ type was not an independent predictor factor in diagnosing LSIL or HSIL/carcinoma. A prospective multicenter study by Luyten et al. compared the distribution of TZ types among women according to age [18]. In their study, type 3 TZ was most commonly reported in women >50 years old (70%). However, there was evidence of a heterogeneous distribution of TZ types 1 and 2 between the age groups, possibly because of a diagnostic uncertainty between these types. They proposed a more precise anatomic distinction between types 1 and 2 in the IFCPC terminology to improve the utility of the new IFCPC classification and to encourage standardized universal reporting of findings. However, the 2017 American Society for Colposcopy and Cervical Pathology (ASCCP) colposcopy standards [19] did not divide cervical TZ into types 1, 2, and 3, mainly because of the poor reproducibility of type 2 TZ in actual clinical work. With regard to cervical TZ, more clinical studies are needed to confirm its feasibility and clinical value in colposcopic examination.

Lesion size has also been included in the new terminology system. Our study revealed a linear trend between lesion size and severity of the diagnosis, and a larger lesion size was more closely related to HSIL+. However, binary logistic regression analysis revealed that lesion size was not an independent predictor factor in diagnosing HSIL+. Several previous studies have shown that a large lesion size was more closely related to HSIL or more, and a small lesion size predicted the absence of a squamous epithelial lesion/CIN [17, 20, 21].

The 2011 IFCPC terminology has revised the grading nomenclature by adding two new pathognomonic, objective colposcopic criteria: the “inner border sign” and “ridge sign”. We analyzed these specific signs in detail, and the specificity and PPV of both signs were high. Other studies have confirmed the clinical utility of these criteria and their strong association with HSIL [22–25]. With regard to other *minor changes* and *major changes*, the incidence of a thin acetowhite epithelium was the highest (65.4%). However, a thin acetowhite epithelium was found in only 27.1% of cases with HSIL, and had little or no association with HSIL+. Some studies have reported that a thin acetowhite epithelium does not suggest the existence of a cervical vaginal lesion [17]; however, other studies have reported a thin acetowhite epithelium with HSIL in an atrophic area [26]. Massad et al. proposed that all acetowhite lesions should be biopsied to maximize the sensitivity of colposcopic diagnosis while maintaining a good specificity [16]. In the present study, we found that two or more colposcopic minor changes were independent predictor factors in diagnosing LSIL. Additionally, we also found that a dense aceto-white epithelium, coarse mosaic, and coarse punctation had a high specificity and PPV for HSIL+ and were significantly associated with severe cervical lesions, and two or more colposcopic major changes were independent predictor factors in diagnosing HSIL+. These findings further confirm that the categorization of major changes and minor changes is appropriate.

Another common sign, a non-stained lesion, was once classified as a major change in the RCI scoring system and previous IFCPC colposcopic terminology. However, non-stained lesions are currently classified as a non-specific sign in the 2011 IFCPC terminology. Our study further confirmed that the diagnostic value of non-stained lesions for HSIL+ is very low, and was not an independent predictor factor for HSIL+; although iodine-stained lesions had a high specificity, PPV, and NPV, the sensitivity was very low. Leukoplakia had a considerable PPV of 53.3% for HSIL+; however, our data revealed that leukoplakia was not an independent predictor factor for HSIL+. The present published studies are discordant on the significance of this terminology. In addition, it is worth noting that 30 of 37 cases suggestive of condyloma were related to LSIL/HSIL, suggesting that we should pay close attention to cervical condyloma during colposcopy, and that cervical biopsy should be performed if indicated.

High risk HPV- related infections are a significant source of cervical morbidity and mortality. Our data revealed that HPV type 16 was identified most frequently, followed by HPV types 52, 58, 56, 33, and 18. The prevalence of HPV type 16 increased with the grade of the cervical disease as defined by biopsy-histological diagnosis, and the presence of HPV type 16 was an independent factor for predicting cervical HSIL+ development, whereas the presence of HPV types 18, 33, 52, 56, and 58 were not associated with this risk. This implies that HR-HPV infections, especially HPV type 16, increase the risk of developing severe lesions. As colposcopy is inadequate for patients or in the case of suspected cervical HSIL+, colposcopic examination should be combined with cervical cytology and HR-HPV genotyping, especially with HPV type 16, to further improve diagnostic accuracy.

Our study had several limitations. First, the study design was retrospective in nature and enrollment bias may have occurred. Second, colposcopy diagnosis may have been biased by prior knowledge of the cervical cytology results. Third, although the conization/hysterectomy data had been used to strengthen the pathology diagnosis, the sample size is less. Finally, we did not compare the overall diagnostic accuracy of 2011 IFCPC criteria with that of other colposcopic diagnostic criteria (such as RCI) for the detection of high-grade CIN.

The clinical application of the 2011 IFCPC terminology is in its preliminary stage. Our data revealed that the categorization of major changes and minor changes is appropriate; the accuracy of colposcopic diagnosis using the IFCPC terminology was stable and did not vary with lesion severity. However, there was only a moderate strength of agreement between colposcopic diagnosis and cervical pathology, and the diagnostic accuracy remains unsatisfactory. Therefore, we propose that colposcopic findings should be combined with cervical cytology and HR-HPV genotyping test, especially with HPV type 16, to further improve the diagnostic accuracy. Studies evaluating the 2011 IFCPC colposcopic terminology are limited and further prospective studies are needed. In future studies, we aim to investigate RCI and 2011 IFCPC criteria and obtain data with respect to accuracy of the two colposcopic diagnostic methods.

## MATERIALS AND METHODS

### Subjects

We retrospectively reviewed the medical records of women who underwent colposcopic examinations and cervical biopsy at the colposcopy clinic of the General Hospital of Tianjin Medical University between September 2012 and September 2016. These women had no previous surgical procedures of the cervix, no history of pelvic radiation or hysterectomy, no sexual intercourse for 3 days before the examination, and no confirmed or clinically suspected immunosuppression (e.g., HIV, corticosteroids, chemotherapy) or other chronic disease that might have compromised their immune system. Colposcopy indications included atypical or abnormal cervical cytology results and/or positivity for HR-HPV and/or suspicious clinical findings. Positive cervical cytology included atypical squamous cells of uncertain significance; atypical squamous cell, cannot exclude HSIL; LSIL; HSIL; atypical glandular cells; and invasive cervical cancer. HPV genotyping was performed via the PCR-reverse dot hybridization method. Eighteen HR-HPV types (16, 18, 31, 33, 35, 39, 45, 51, 52, 53, 56, 58, 59, 66, 68, 73, 82, and 83) and five LR-HPV types (6, 11, 42, 43, and 81) were identified. The data in this study were collected from the hospital’s archived database. This study did not influence the diagnosis or treatment of the patients. Informed consent was waived by the ethics review board of the General Hospital of Tianjin Medical University. The institutional review board approved this (No. IRB 2016-YX-073).

### Colposcopy and biopsy procedure

At the colposcopy clinic, all colposcopies were performed by two expert colposcopists with more than five years of experience who had received the ASCCP professional colposcopy training. All colposcopic examinations were performed and recorded using the VIZ-YD system, which is a video exoscope-based system (optical electronic integration colposcopy, Beijing SWSY technology Co., Ltd, China) that allows full HD video documentation of the colposcopic examination and is used for clinical records and research. Steps in colposcopic assessment of the cervix included the following: 1) clean the cervix with normal saline; 2) assess the cervix about 1 min after the application of 5% acetic acid, and observe margins of the lesion, epithelial color and vascular patterns; and 3) assess the cervix after the application of diluted Lugol’s iodine solution, and observe iodine staining. Colposcopic findings were described using the 2011 IFCPC terminology: (1) TZ types, lesion size in percentage of cervix, and normal colposcopic findings (such as original squamous epithelium: mature or atrophic, columnar epithelium ectopy, Nabothian cysts, crypt [gland] openings); (2) abnormal colposcopic findings, grade 1( minor) (thin acetowhite epithelium[irregular, geographic, border], fine punctation, fine mosaic), grade 2 (major) (dense aceto-white epithelium, rapid appearance of acetowhitening, cuffed crypt [gland] openings, coarse punctation, coarse mosaic, sharp border, inner border sign, ridge sign), non specific (Leukoplakia [keratosis, hyperkeratosis], Erosion, stained/non-stained Lugol’s staining [Schiller’s test]); (3) suspicious for invasion, atypical vessels, additional signs; and (4) miscellaneous findings, such as condyloma, polyp, inflammation, and post treatment consequence. It is impossible that all abnormal findings (suggestive of the same grade disease) simultaneously exist in one patient. As a result, colposcopists made a preliminary colposcopic diagnosis for each colposcopic finding on the basis of the criteria of the IFCPC. It should be noted that the same colposcopic finding could be present in different grades of lesions. When doubtful diagnosis occurred, all the inconsistent diagnoses were analyzed by another senior colposcopist, who made a decision on the final colposcopic diagnosis.

All colposcopically detected abnormal areas were biopsied, and large or multiple lesions were subjected to more than one biopsy. If the colposcopic examination showed no lesion in the cervix, a random biopsy specimen was obtained at the squamocolumnar junction in that quadrant at 3, 6, 9, or 12 o’clock. An endocervical curettage was performed after the cervical biopsy.

All biopsy specimens were fixed in formaldehyde and embedded in paraffin according to the routine procedures of the pathology department. The biopsy specimens were examined by the pathologist. After examining the specimen, an experienced expert pathologist made a final diagnosis according to the 2012 Lower Anogenital Squamous Project’s two-tier squamous intraepithelial lesions (SIL) terminology (LSIL and HSIL). In uncertain cases, P16 immunohistochemistry should be used in the assessment of HSILs, particularly in dealing with a specimen that already has an H&E morphologic interpretation of HSIL(CIN 2) and in the distinction between HSIL(CIN2 or CIN3) and its mimics, such as immature squamous metaplasia, atrophy, reparative epithelial changes, and tangential cutting [7]. LSIL refers to koilocytosis, flat condyloma, CIN 1, or P16 negative CIN2; HSIL refers to CIN3 or P16 positive CIN2 [8]. A pathology slide review was not performed.

### Statistical methods

SPSS 20.0 software (SPSS, Inc., Chicago, IL) was used for statistical analysis. The significance of agreement between the colposcopic diagnosis and cervical pathology was assessed with the chi-square statistic (χ^2^ test) and weighted kappa values. The sensitivity, specificity, PPV, NPV, and Youden index were used to compare the colposcopic diagnosis and cervical pathology. P-values < 0.05 were considered statistically significant.

## Abbreviations

HSIL: high-grade squamous intraepithelial lesion
IFCPC: International Federation for Cervical Pathology and Colposcopy
LSIL: low-grade squamous intraepithelial lesion
MIC: micro invasive cervical cancer
NPV: negative predictive value
PPV: positive predictive value
RCI: Reid colposcopic index
TZ: transformation zone
ASCCP: American Society for Colposcopy and Cervical Pathology
